# Development and application of a fast and efficient CRISPR-based genetic toolkit in *Bacillus amyloliquefaciens* LB1ba02

**DOI:** 10.1186/s12934-022-01832-2

**Published:** 2022-05-28

**Authors:** Qinglong Xin, Yudan Chen, Qianlin Chen, Bin Wang, Li Pan

**Affiliations:** School of Biology and Biological Engineering, Guangzhou Higher Education Mega Centre, South China University of Technology, Panyu District, Guangzhou, 510006 Guangdong People’s Republic of China

**Keywords:** *B. amyloliquefaciens*, CRISPR/Cas9n-AID, Base editing, α-amylase

## Abstract

**Background:**

*Bacillus amyloliquefaciens* is generally recognized as food safe (GRAS) microbial host and important enzyme-producing strain in the industry. *B.amyloliquefaciens* LB1ba02 is a production strain suitable for secreting mesophilic α-amylase in the industry. Nevertheless, due to the low transformation efficiency and restriction-modification system, the development of its CRISPR tool lags far behind other species and strains from the genus *Bacillus*. This work was undertaken to develop a fast and efficient gene-editing tool in *B.amyloliquefaciens* LB1ba02.

**Results:**

In this study, we fused the nuclease-deficient mutant Cas9n (D10A) of Cas9 with activation-induced cytidine deaminase (AID) and developed a fast and efficient base editing system for the first time in *B. amyloliquefaciens* LB1ba02. The system was verified by inactivating the *pyrF* gene coding orotidine 5'-phosphate decarboxylase and the mutant could grow normally on M9 medium supplemented with 5-fluoroorotic acid (5-FOA) and uridine (U). Our base editing system has a 6nt editing window consisting of an all-in-one temperature-sensitive plasmid that facilitates multiple rounds of genome engineering in *B. amyloliquefaciens* LB1ba02. The total editing efficiency of this method reached 100% and it achieved simultaneous editing of three loci with an efficiency of 53.3%. In addition, based on the base editing CRISPR/Cas9n-AID system, we also developed a single plasmid CRISPR/Cas9n system suitable for rapid gene knockout and integration. The knockout efficiency for a single gene reached 93%. Finally, we generated 4 genes (*aprE*, *nprE*, *wprA*, and *bamHIR*) mutant strain, LB1ba02△4. The mutant strain secreted 1.25-fold more α-amylase into the medium than the wild-type strain.

**Conclusions:**

The CRISPR/Cas9n-AID and CRISPR/Cas9n systems developed in this work proved to be a fast and efficient genetic manipulation tool in a restriction-modification system and poorly transformable strain.

**Supplementary Information:**

The online version contains supplementary material available at 10.1186/s12934-022-01832-2.

## Background

*Bacillus amyloliquefaciens* is generally recognized as food safe (GRAS) microbial host and an important industrial strain in the production of α-amylase and proteases [1]. Besides, it can secrete antibacterial substances to inhibit the activity of fungi and bacteria [2–5]. As a closely related species of *Bacillus subtilis*, *B. amyloliquefaciens* can be used to produce various enzyme preparations [1, 6–8]. *B. amyloliquefaciens* LB1ba02 is an important production strain suitable for secreting mesophilic α-amylase in the industry. However, the extracellular proteolytic enzyme secreted by undomesticated strains hydrolyzes the protein of interest to varying degrees. Moreover, the restriction-modification system existed in *B. amyloliquefaciens* leads to the degradation of the exogenous DNA to be transformed [9]. The low transformation efficiency and the restriction-modification system hinder the development of its gene-editing tool.

The traditional non-CRISPR gene-editing method of *B. amyloliquefaciens* is through a two-step homologous recombination procedure, which uses a thermosensitive rolling-circle replication plasmid [10]. However, this method has a low probability of double-crossover when performing the second recombinational event. This makes researchers need to consume a lot of time and energy to carry out screening work. To increase the probability of the second recombination event, Zhang et al. [11] used the *upp* gene as a counterselectable marker and added 5-fluorouracil (5-FU) as a growth pressure to select for double-crossover recombinants [11]. Under the growth pressure of 5-FU, the probability of the second homologous replacement was greatly improved. Nevertheless, the entire process of two-step homologous recombination, including gene editing and plasmid curing, still takes too long. In addition, it cannot achieve multiple gene knockouts simultaneously.

With the development of genome editing technology, the discovery of the CRISPR system has been endowed with epoch-making significance [12, 13]. Compared with traditional homologous recombination editing, the CRISPR/Cas9 system is more efficient and faster. Furthermore, it can disrupt multiple genes simultaneously, reducing the workload of gene editing significantly [13, 14]. The CRISPR/Cas9 system has been implemented in *Escherichia coli*, *Bacillus licheniformis*, and *B. subtilis*, etc. [15–18]. The CRISPR-based genetic tools developed by other species and strains from the genus *Bacillus* can be adapted to *B. amyloliquefaciens* theoretically; however, it needs to be further verified through experiments due to the distinctions between different species. Qiu et al. [19] reported for the first time that a dual plasmid CRISPR/Cas9n system could achieve the disruption of the *B. amyloliquefaciens* NB gene by integrating the Cas9n protein and sgRNA into two different vectors [19]. Zhao et al. [20] further fused the nuclease-deficient mutant dCas9 of Cas9 with the ω subunit of RNA polymerase achieved gene transcription regulation in *B. amyloliquefaciens* 205 [20]. To date, the CRISPR/Cas9 system is the only CRISPR-based genetic tool reported in *B. amyloliquefaciens*. When performing gene editing by CRISPR/Cas9, donor DNA needs to be introduced to prevent Cas9 from causing genomic double-strand breaks (DSBs) and bacterial death, unfavorable for the simultaneous knockout of multiple genes, especially in *B. amyloliquefaciens* [21, 22]. Nishida et al. [23] developed the Cas9n-AID base editor by fusing AID from sea lamprey with Cas9n [23]. The Cas9n-AID base editor performs direct conversion of specific Cs into Ts in a programmable manner that does not require DNA DSBs or the introduction of donor DNA templates and is, therefore, more suitable for simultaneous editing of multiple genes [24, 25].

At present, the Cas9n(dCad9)-AID base editor has been successfully developed and applied in different prokaryotes with high efficiency, such as *Escherichia coli*, *Corynebacterium glutamicum*, *Streptomyces* and *Agrobacterium* [25–28]. Wang et al. [29] showed that Target-AID base editing could not only achieve C-T conversion, but had a certain probability to achieve C-A or C-G conversion in bacteria [29]. In addition, Shelake et al. [30] recently developed different variants of CBEs (PmCDA1, evoCDA1, APOBEC3A) and ABEs (ABE8e, ABE9e) and successfully applied them to bacteria [30]. Whereas different base editing tools have been used in prokaryotes as well, it has not been developed in the *B. amyloliquefaciens* yet.

In this study, we adapted the CRISPR/Cas9n-AID and implemented the base editing system in *B. amyloliquefaciens* LB1ba02. The CRISPR/Cas9n-AID base editing system has a 6nt editing window and the total editing efficiency reached 100%. In addition, this method achieved simultaneous editing of three loci with an efficiency of 53.3%. Based on the CRISPR/Cas9n-AID system, we also developed a single plasmid CRISPR/Cas9n system suitable for rapid gene knockout and integration. Finally, we obtained a mutant *B. amyloliquefaciens* strain, LB1ba02△4, with the deletion of 4 genes (*aprE*, *nprE*, *wprA*, and *bamHIR*). LB1ba02△4 strain secreted 1.25-fold more α-amylase into the medium than the wild strain. The single plasmid CRISPR/Cas9n-AID and CRISPR/Cas9n systems developed in this work expand the range of the genetic manipulation of *B. amyloliquefaciens.*

## Results

### CRISPR/Cas9n mediated cytosine deaminase base editing in *B. amyloliquefaciens* LB1ba02

**Fig. 1:**
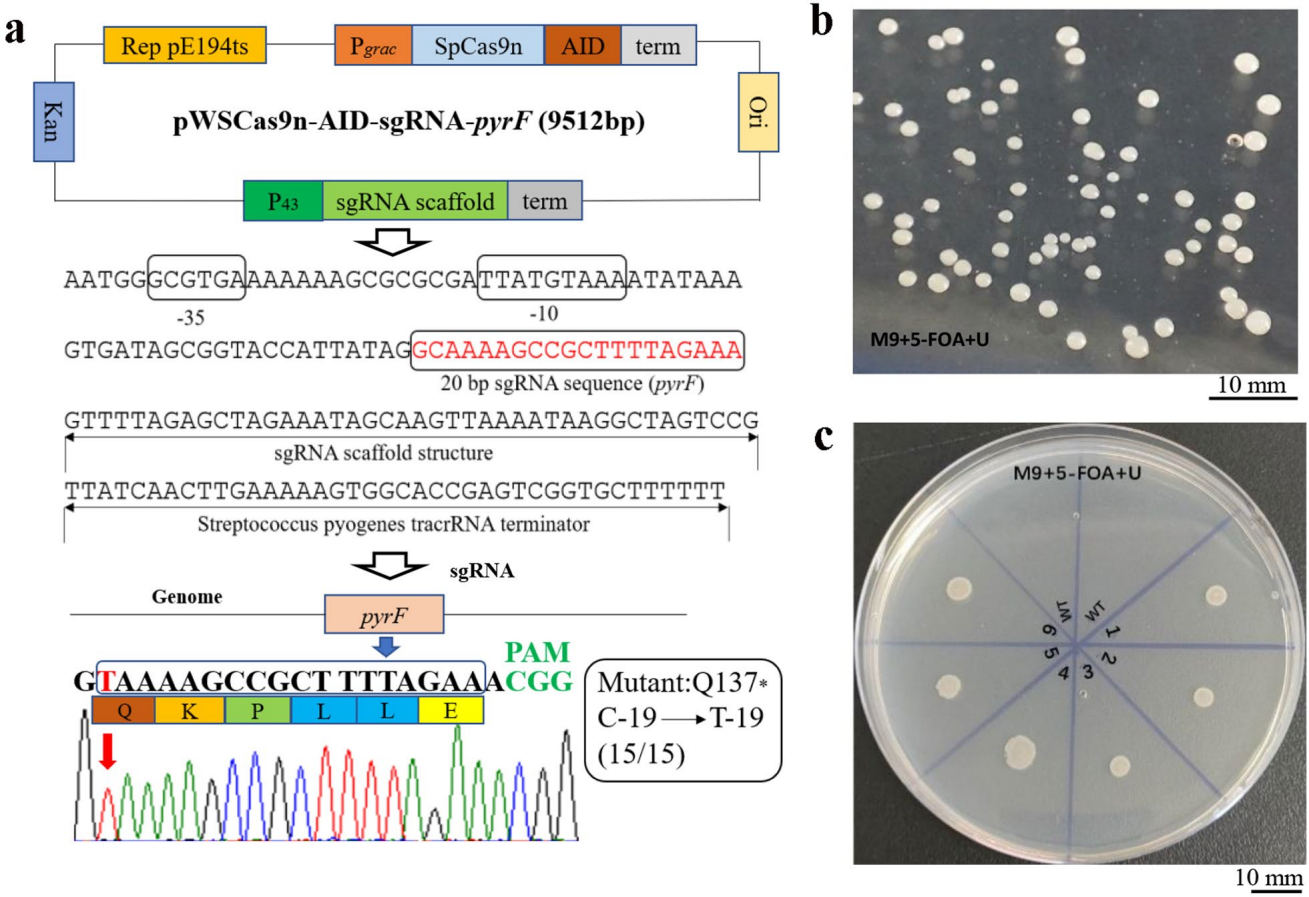
CRISPR/Cas9n-AID base editing system in *B. amyloliquefaciens* LB1ba02. **a** Physical map of plasmid pWSCas9n-AID-sgRNA-*pyrF* containing the *E. coli* replication origin, the *Bacillus* replication origin Rep pE194^ts^, a kanamycin resistance gene, and the AID fused with Cas9n under the control of IPTG-inducible promoter P_*grac*_. The sequence of a synthetic sgRNA module containing P_43_ promoter, 20 bp sgRNA sequence of *pyrF*, SpCas9n binding scaffold, and *Streptococcus pyogenes* tracrRNA terminator. **b** The cell suspensions were diluted to 10^–3^ and spread on an M9 plate containing 5-FOA and uridine. **c** The wild-type and base editing mutant strains (LB1ba02△*pyrF*) were cultivated for 24 h at 37 °C on M9 agar plate with 5-FOA and uridine. WT: wild-type strain of LB1ba02; 1,2,3,4,5,6: *pyrF* deletion strains LB1ba02△*pyrF*

To test whether CRISPR/Cas9n-AID base editing system can be used in *B. amyloliquefaciens* LB1ba02, we selected the *pyrF* gene encoding orotidine 5ʹ-phosphate decarboxylase as a target. The mutant strain with the inactive *pyrF* gene is auxotrophic for U [31–34]. The Cas9n (D10A) protein with AID ortholog (PmCDA1 from sea lamprey) was fused and expressed under the control of the IPTG-inducible promoter P_*grac*_. P_43_ is a constitutive strong promoter responsible for transcription of the cytidine deaminase gene from *B. subtilis*, which can continuously express genes in logarithmic growth phase and stationary phase. Therefore, it was designed for the transcription of sgRNA. The base editing plasmid pWSCas9n-AID-sgRNA-*pyrF* was constructed as shown in Fig. 1a. The plasmid was transformed into *B. amyloliquefaciens* LB1ba02, spread on kanamycin containing 50 μg/mL LB plate and cultured at 30 °C for 12–16 h. It is worth mentioning that due to the large single temperature-sensitive plasmid and the toxicity of Cas9n protein to bacteria, this will result in low transformation efficiency of pWSCas9n-sgRNA plasmid (1–10 transformants/μg normally). The correct single transformant containing the plasmid pWSCas9n-AID-sgRNA-*pyrF* was induced in a liquid medium with 50 μg/mL kanamycin and 100 μM IPTG at 30 °C, 220 rpm for 24 h. Then the cell suspensions were diluted to 10^–3^ and spread on an M9 plate containing 5-FOA and U (Fig. 1b). 15 single colonies on the plate were selected randomly and target gene was amplified by the VF-(*pyrF*)-F/R primers; further, verified by DNA sequencing. The results showed that the *pyrF* genes in the selected single colonies were edited; thus, the efficiency reached 100% under the selection pressure of 5-FOA. The *pyrF* gene deletion strain and the wild strain were inoculated into the minimal medium M9 supplemented with 5-FOA and U. The mutant strain grew normally; however, 5-FOA was lethal to the wild strain (Fig. 1c). This result indicates that the CRISPR/Cas9n-AID system can successfully perform base editing in *B. amyloliquefaciens* LB1ba02.

### Base editing window of the CRISPR/Cas9n-AID system in *B. amyloliquefaciens* LB1ba02

**Fig. 2:**
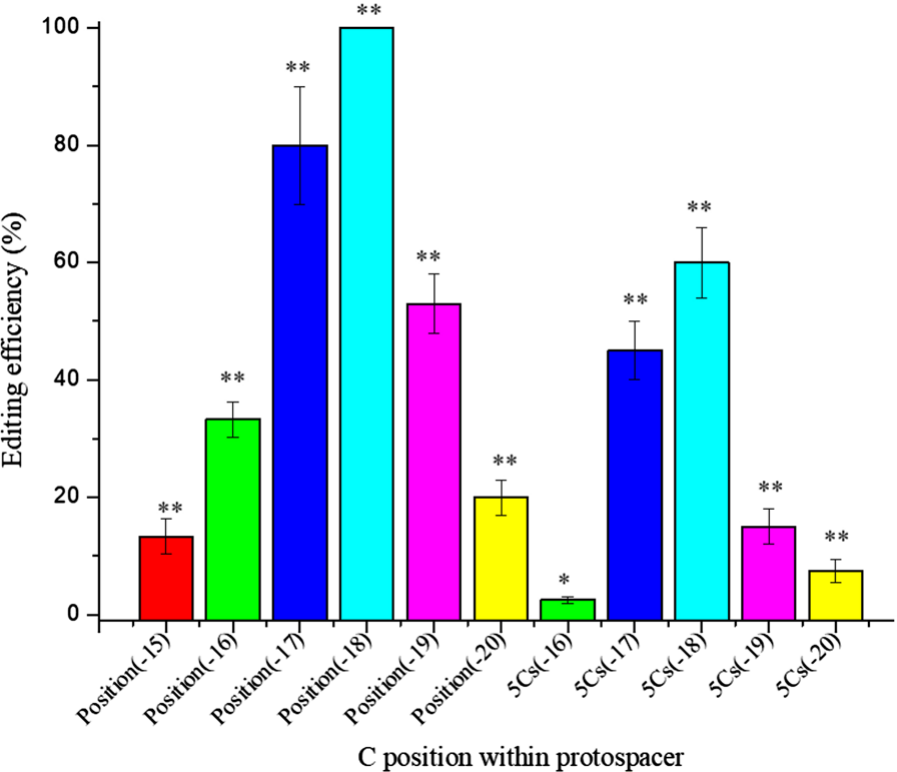
Editing efficiency determination of cytosine at the − 15 to − 20 positions. The means of three independent technical replicates are presented; error bars represent standard deviations. * *P* < 0.05 and ** *P* < 0.01 compared with control without presence of pWSCas9n-AID-sgRNA

The CRISPR/Cas9n-AID base editing system has been reported to target − 16 to − 20 positions upstream the PAM sequence [23, 25]. However, we found that this system has a 6 nt editing window (− 15 to − 20 positions upstream the PAM sequence) in *B. amyloliquefaciens* LB1ba02. We designed 6 different sgRNAs to test the editing efficiency of each position (Additional file 1: Fig. S8). The results showed that the − 18 position had the highest editing efficiency of 100% and the − 17 position had the editing efficiency of 80%. The editing efficiency of other loci was gradually decreasing. The editing efficiency of − 19 and − 16 positions were 53.3% and 33.3%, respectively. However, the − 20 and − 15 positions were only 20% and 13.3%, respectively (Fig. 2).

With the deepening of the research, we found that the editing efficiency of each position was affected by neighboring cytosines. The targeted sgRNA located in the *abnA* gene (arabinan-endo 1, 5-alpha-L-arabinase) containing five consecutive cytosines (5Cs) from positions − 16 to − 20 upstream of the PAM was selected to investigate the discrepancy (Additional file 1: Fig. S9). The editing efficiency of the 5Cs window was subsequently tested. The editing efficiencies of the − 17 and − 18 positions were 46.6% and 60%, respectively. The editing efficiency of − 19 and − 20 positions were only 13.3% and 6.6%, respectively. The − 16 position had the lowest efficiency, only 2.2% (Fig. 2). The results showed that the overall editing efficiency of each locus declined due to the influence of adjacent cytosines. Nevertheless, the − 17 and − 18 positions maintained the highest editing efficiency.

### CRISPR/Cas9n-AID mediated multi-gene editing

**Fig. 3:**
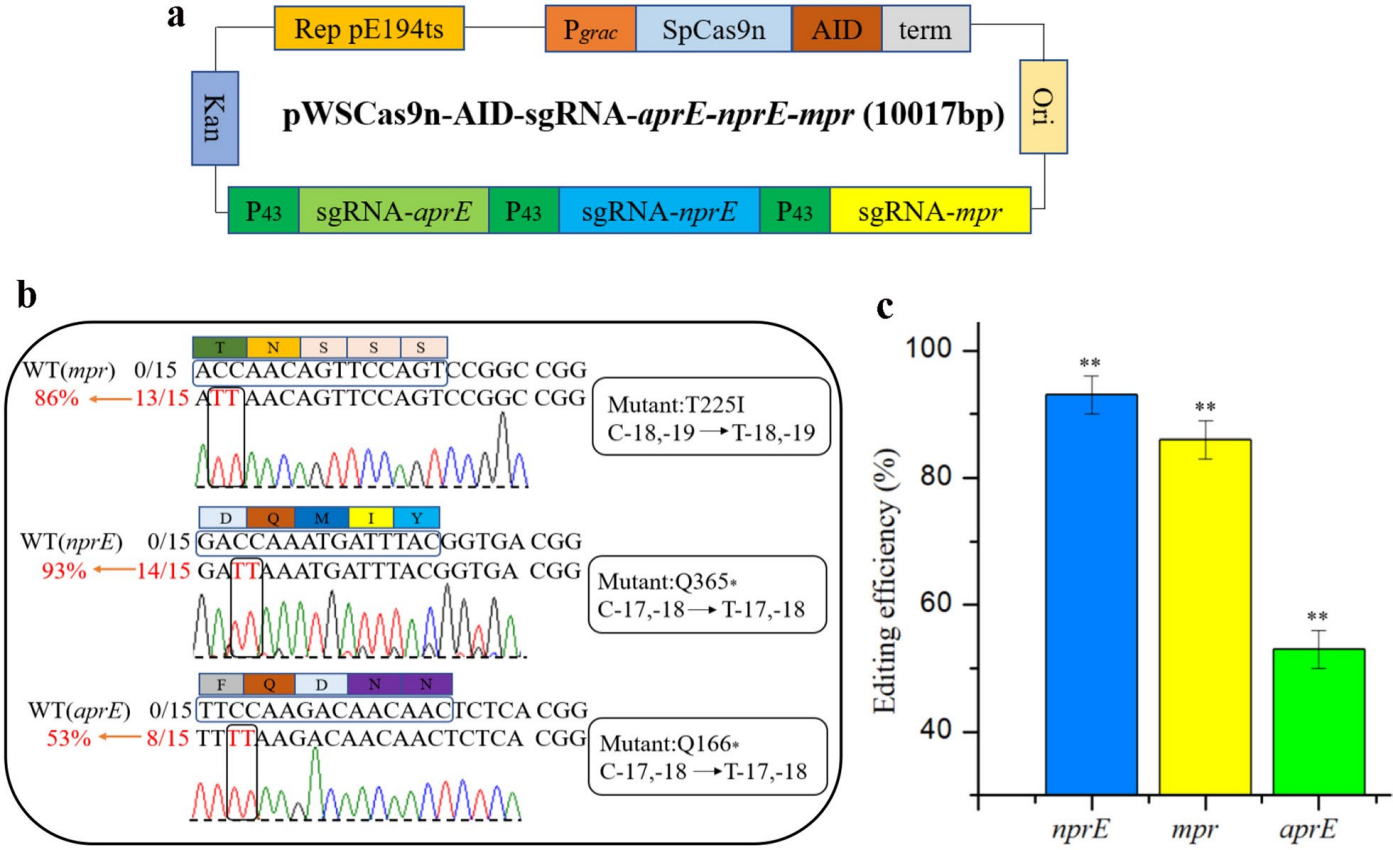
Editing of multiple genes by the CRISPR/Cas9n-AID system simultaneously. **a** Physical map of the edited plasmid pWSCas9n-AID-sgRNA-*aprE-nprE-mpr*, which three P_43_-sgRNAs were connected in series and inserted into the backbone plasmid pWSCas9n-AID. **b**, **c** Three genes of *aprE*, *nprE*, and *mpr* were simultaneously edited and the editing efficiency was verified by DNA sequencing. The means of three independent technical replicates are presented; error bars represent standard deviations. ** *P* < 0.01 compared with control without presence of pWSCas9n-AID-sgRNA

To verify whether the CRISPR/Cas9n-AID base editing system can achieve multi-gene editing in *B. amyloliquefaciens* LB1ba02, we selected three extracellular protease genes *aprE*, *nprE*, and *mpr* for testing. Three P_43_-sgRNAs were connected in series and inserted into the pWSCas9n-AID plasmid to obtain the edited plasmid pWSCas9n-AID-sgRNA-*aprE-nprE-mpr* (Fig. 3a). The resulted plasmid was transformed into the wild strain LB1ba02, spread on kanamycin containing 50 μg/mL LB plate and cultured at 30 °C for 12–16 h. The correct single transformant containing the plasmid pWSCas9n-AID-sgRNA-*aprE-nprE-mpr* was induced in a liquid medium with 50 μg/mL kanamycin and 100 μM IPTG at 30 °C, 220 rpm for 24 h. Then the cell suspensions were diluted to 10^–5^ and spread on the LB plate at 37 °C for 12 h. 15 single colonies on the plate were selected randomly and target genes were amplified by the VF-(*aprE*)-F/R, VF-(*nprE*)-F/R, and VF-(*mpr*)-F/R primers. DNA sequencing was performed to determine the editing efficiency of the *aprE*, *nprE*, and *mpr* genes. As shown in Fig. 3b, c, *aprE*, *nprE*, and *mpr* sites were simultaneous edited with an efficiency of 53.3%. The editing efficiency of *aprE*, *nprE* and *mpr* sites were 53.3%, 93.3% and 86.7%, respectively. In addition, the conversion of stop codons at the *aprE* and *nprE* sites was achieved so that these two genes were knocked out, and the resulting strain was named LB1ba02△2.

### The single plasmid CRISPR/Cas9n system

**Fig. 4:**
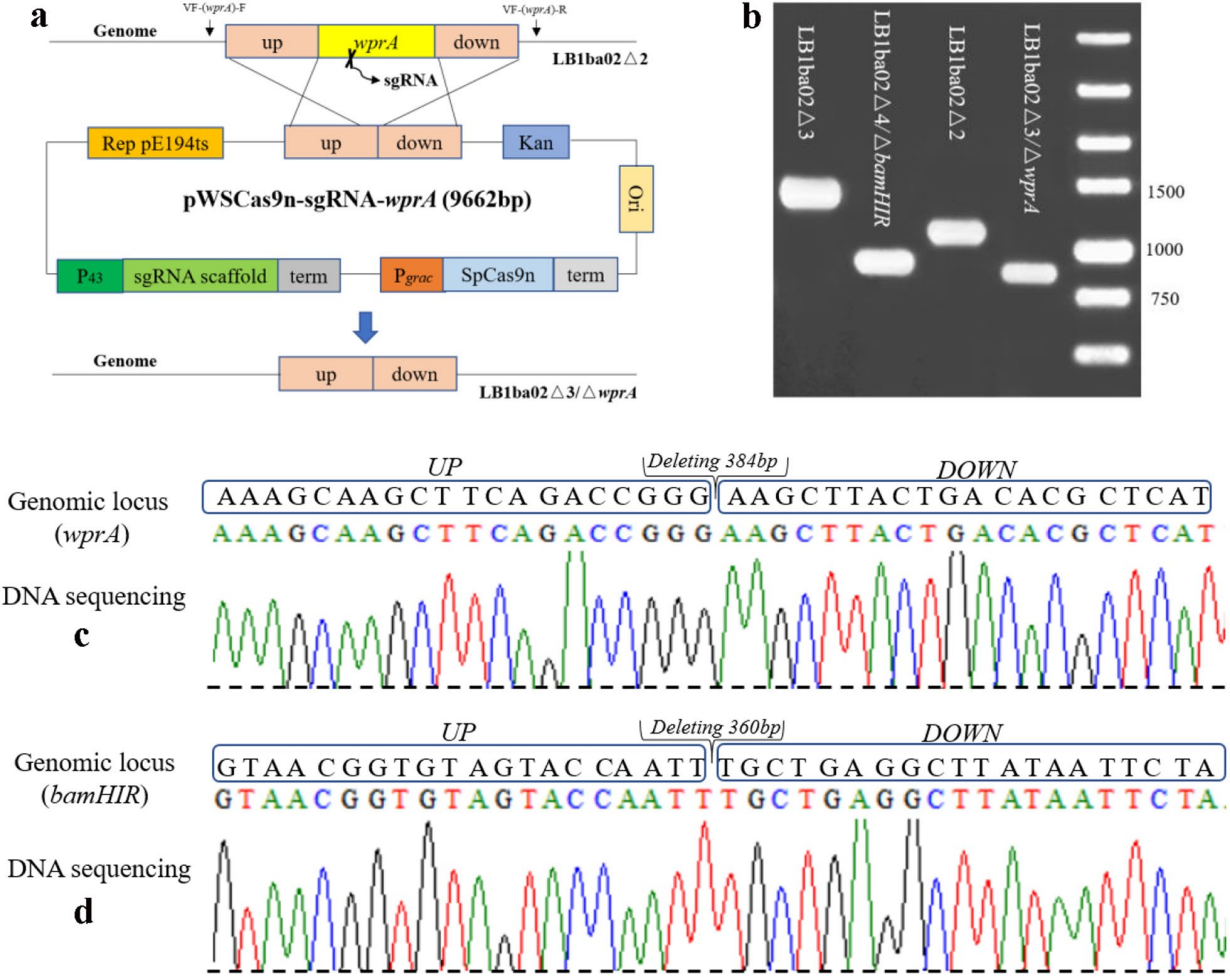
The single plasmid CRISPR/Cas9n system. **a** Physical map of plasmid pWSCas9n-sgRNA-*wprA* containing the *E. coli* replication origin, the *Bac*illus replication origin Rep pE194^ts^, a kanamycin resistance gene, Cas9n under the control of IPTG-inducible promoter P_*grac*_, the sgRNA transcribed from the *B. subtilis* promoter P_43_, and donor DNA was used to repair SSB. **b** The agarose gel electrophoresis verification of *wprA* and *bamHIR* deletions by the single plasmid CRISPR/Cas9n system. **c** DNA sequencing of *wprA* deletion, losing 384 bp*.* **d** DNA sequencing of *bamHIR* deletion, losing 360 bp

In the backbone plasmid pWSCas9n constructed in this work, rapid gene knockout and integration can be achieved when the P43-sgRNA expression cassette and homologous repair template are inserted. The *wprA* gene cannot be knocked out by traditional homologous recombination [35]; thus, it was selected to demonstrate the CRISPR/Cas9n system's editing efficiency. The knockout plasmid pWSCas9n-sgRNA-*wprA* was transformed into LB1ba02△2, spread on kanamycin containing 50 μg/mL LB plate and cultured at 30 °C for 12–16 h. The correct single transformant containing the plasmid pWSCas9n-sgRNA-*wprA* was induced in a liquid medium with 50 μg/mL kanamycin and 100 μM IPTG at 30 °C, 220 rpm for 24 h. Then the cell suspensions were diluted to 10^–5^ and spread on the LB plate at 37 °C for 12 h. 15 single colonies on the plate were subsequently selected randomly and target gene was amplified by the VF-(*wprA*)-F/R primers located in the genome outside of the homologous recombination flanks (Fig. 4a); further, verified by DNA sequencing. As shown in Fig. 4b, c, the *wprA* gene was deleted successfully, resulting in the LB1ba02△3 strain, and 93% editing efficiency was reached after IPTG induction (Additional file 1: Figure S2). To reduce the pretreatment step of *Bam*HI methylase before exogenous plasmid transformation, we further disrupted the restriction endonuclease gene *bamHIR* (Fig. 4b–d) and obtained LB1ba02△4 strain. The transformation and editing process of knockout plasmid pWSCas9n-sgRNA-*bamHIR* refers to pWSCas9n-sgRNA-*wprA*. After knocking out the *bamHIR* gene, the exogenous DNA plasmid with the *Bam*HI restriction site could be transformed into LB1ba02△4 without methylation treatment (Additional file 1: Figure S3).

### Gene integration by the single plasmid CRISPR/Cas9n system

**Fig. 5:**
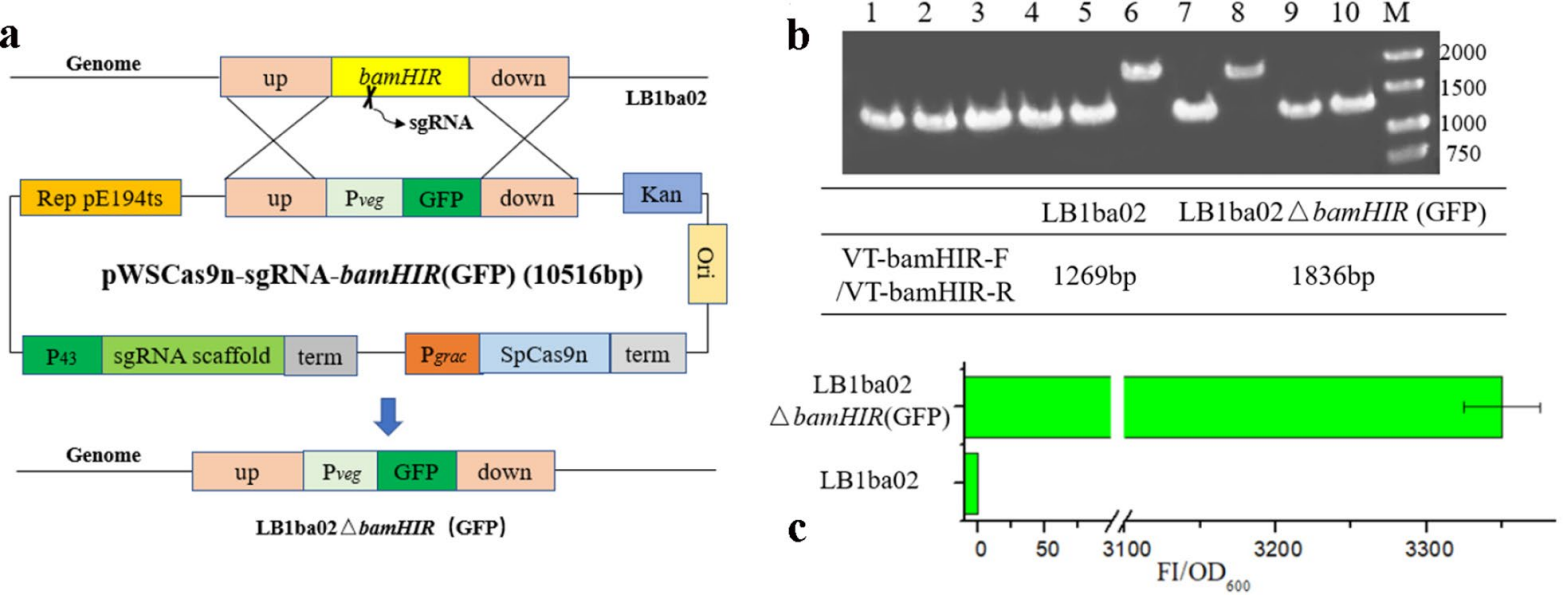
Gene integration by the CRISPR/Cas9n system. **a** Physical map of integration plasmid pWSCas9n-sgRNA-*bamHIR*(GFP). **b** The agarose gel electrophoresis verification of P_*veg*_-GFP integration by the single plasmid CRISPR/Cas9n system. **c** Fluorescence intensity measurement of LB1ba02 and LB1ba02△*bamHIR*(GFP)

Although the base-editing system can achieve C − T base conversion, it cannot achieve gene integration. Contrary, the CRISPR/Cas9n system can achieve gene integration [36, 37]. To determine whether our system can achieve gene integration efficiently, we selected the *bamHIR* site for testing. As shown in Fig. 5a, a fluorescent protein integration plasmid pWSCas9n-sgRNA-*bamHIR*(GFP) was constructed and transformed into *B. amyloliquefaciens* LB1ba02. Then spread on kanamycin containing 50 μg/mL LB plate and cultured at 30 °C for 12–16 h. The correct single transformant containing the plasmid pWSCas9n-sgRNA-*bamHIR*(GFP) was induced in a liquid medium with 50 μg/mL kanamycin and 100 μM IPTG at 30 °C, 220 rpm for 24 h. Then the cell suspensions were diluted to 10^–5^ and spread on the LB plate at 37 °C for 12 h. Subsequently, 10 single colonies on the plate were selected randomly and target gene was amplified by the VF-(*bamHIR*)-F/R primers located in the genome outside of the homologous recombination flanks; further, verified by DNA sequencing. The results showed that only 2 of the 10 transformants picked at random were positive, and the gene integration efficiency was 20% (Fig. 5b). The P_*veg*_-GFP-integrated strain LB1ba02△*bamHIR*(GFP) was cultured in a 24-well plate and the fluorescence intensity was measured after 24 h. The wild-type strain LB1ba02 was used as a control. As shown in Fig. 5c, the fluorescence intensity of LB1ba02△*bamHIR*(GFP) reached 3350, while no GFP expression was observed in the LB1ba02 strain.

### Production of α-amylase (AmyX) using *B. amyloliquefaciens* LB1ba02Δ4

**Fig. 6:**
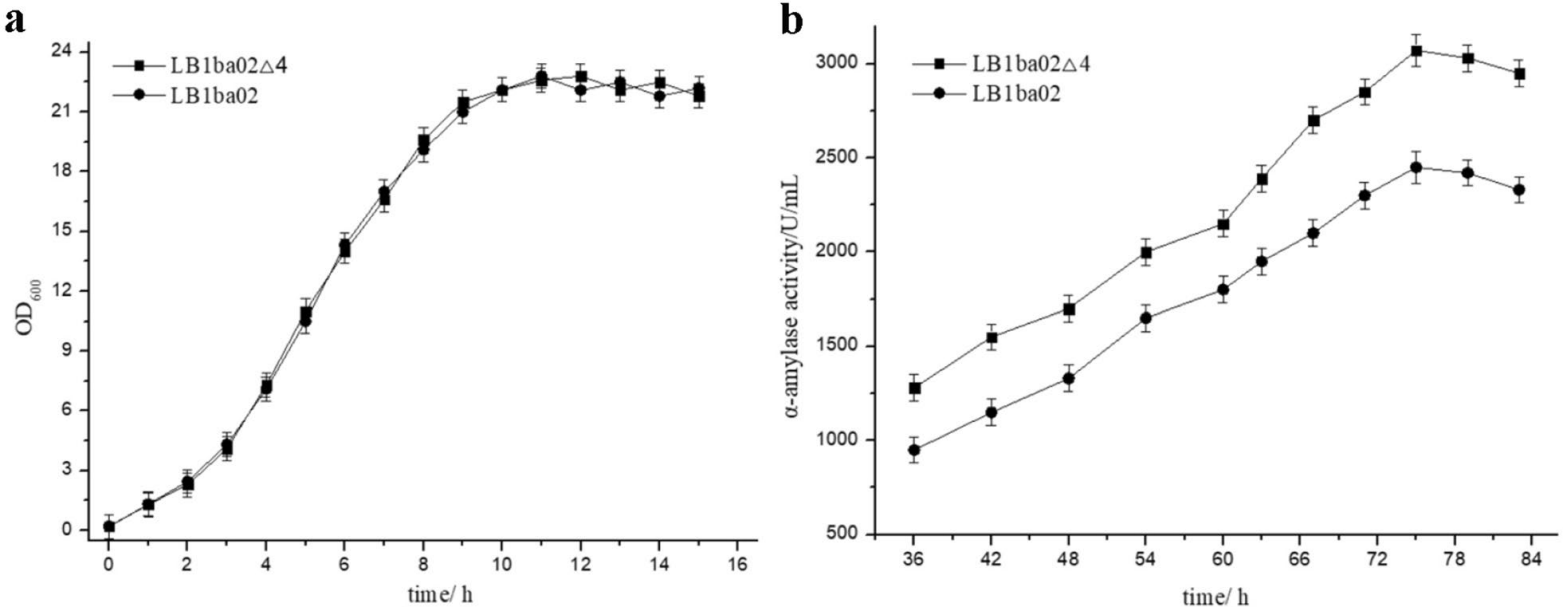
**a** Cell growth curve of LB1ba02 and LB1ba02△4. **b** α-amylase activity assay of LB1ba02 and LB1ba02△4. The means of three independent technical replicates are presented; error bars represent standard deviations

Through the CRISPR/Cas9n-AID and CRISPR/Cas9n systems developed in this work, we successively knocked out three extracellular protease genes (*aprE*, *nprE*, and *wprA*) and one restriction endonuclease gene (*bamHIR*) in *B. amyloliquefaciens* LB1ba02, resulting in strain LB1ba02△4. To evaluate LB1ba02△4 mutation features, cell growth and AmyX secretion were investigated. As shown in Fig. 6a, there were no distinct differences in cell growth (OD_600_) between LB1ba02△4 and LB1ba02, indicating that *aprE*, *nprE*, *wprA*, and *bamHIR* deletion did not influence the biomass. After disruption of the extracellular proteases AprE, NprE, and WprA, the ability of LB1ba02△4 to secrete AmyX was improved. Compared with LB1ba02, the mutant LB1ba02△4 secreted 1.25-fold more AmyX into the medium and the highest enzyme activity reached 3072.54 U/mL in the shake flask fermentation (Fig. 6b). Therefore, LB1ba02Δ4 can be used as a more efficient host to express the protein of interest.

### Plasmid curing

The Rep pE194^ts^ of the pWSCas9n backbone plasmid is a *Bacillus* temperature-sensitive replicon, which replicates normally at a culture temperature of 30 °C. The plasmid will suicide and cannot replicate when the culture temperature exceeds 37 °C. To confirm the curing efficiency of the temperature-sensitive plasmid pWSCas9n-sgRNA-*bamHIR*, we cultured the recombinant strains at 42 °C for 12 h in a liquid medium. The cell suspensions were diluted to 10^–5^ and spread on the LB plate at 37 °C for 12 h. Then 45 colonies were simultaneously spotted to the LB plate containing kanamycin and the non-resistant LB plate at 37 °C for 12 h to observe the plasmid curing efficiency. As shown in Additional file 1: Figure S4, among the 45 screened colonies, 10 colonies did not grow on the kanamycin LB plate, indicating that 22.2% of the plasmid pWSCas9n-sgRNA-*bamHIR* had been removed.

## Discussion

*B. amyloliquefaciens* is generally recognized as food safe (GRAS) microorganism in industry and possesses a powerful ability to secrete extracellular proteins. However, its low transformation efficiency and the restriction-modification system hinder the development of its CRISPR tool. Compared with traditional homologous recombination, CRISPR/Cas9 system gene editing requires shorter time and higher efficiency, and has been widely used in various animals, plants and microorganisms [38–40]. The CRISPR/Cas9 system in *B. amyloliquefaciens* was not reported for the first time until 2020 [19]. However, DSBs in the genome caused by Cas9 are often fatal to bacteria. Therefore, the donor DNA needs to be inserted to repair the DSBs when genetic manipulation is performed through the CRISPR/Cas9 system [21, 41, 42]. Co-transformation can reduce the difficulty of plasmid construction, and it has realized co-transformation of Cas9 plasmid and linear repair fragment donor DNA in *B. subtilis* [43]. Nevertheless, in our actual research process, we found that co-transformation of the pWSCas9n plasmid and the donor DNA fragment did not achieve gene knockout in *B. amyloliquefaciens* LB1ba02 (data not shown). This makes it necessary to insert multiple donor DNAs into the plasmid when the CRISPR/Cas9 system performs simultaneous multi-gene knockout, which will increase the difficulty of constructing recombinant plasmids.

Base editing is a relatively new method of genome editing and a point mutation essentially. Furthermore, it is theoretically possible for the mutations generated by this method to be reverted. To avoid the reverse mutation, Cas9n protein is usually expressed and fused with uracil DNA glycosylase inhibitor (UGI) in microorganism. Of course, with the continuous replication of genome, there is still a very low probability of reverting the mutation under natural continuous subculture conditions after the base has been successfully edited. Compared with CRISPR/Cas9, the single-base editor performs direct conversion of target bases in a programmable manner, without the need for DNA DSBs or the introduction of exogenous templates, and is more suitable for simultaneous editing of multiple genes, especially in *B. amyloliquefaciens* [24, 25]. In this work, we fused AID with Cas9n and implemented the base editing system for the first time in *B. amyloliquefaciens* LB1ba02. In addition, the *pyrF* gene coding orotidine 5'-phosphate decarboxylase as a food-grade auxotrophic reverse screening marker was applied to *B. amyloliquefaciens* LB1ba02, and the editing efficiency reached 100%. Besides, it achieved simultaneous editing of three loci (*aprE*, *nprE*, and *mpr*) with an efficiency of 53.3%. This shows, the single-base editing system can work well in *B. amyloliquefaciens* LB1ba02.

Although the base-editing system can achieve C-T base conversion, it cannot achieve gene integration. Contrary, the CRISPR/Cas9n system can achieve gene integration [36, 37]. The single plasmid CRISPR/Cas9n system developed in this work could perform rapid and efficient gene deletion in *B. amyloliquefaciens* LB1ba02. In addition, we achieved the genomic integration of the green fluorescent protein GFP through the CRISPR/Cas9n system. However, the integration efficiency is only 20%, which still needs to be improved. It is worth mentioning that due to the large single temperature-sensitive plasmid and the toxicity of Cas9n protein to bacteria, this will result in low transformation efficiency of pWSCas9n-sgRNA plasmid (1–10 transformants/μg normally). Thus, the correct single transformant needs to be induced in a liquid medium with 50 μg/mL kanamycin and 100 μM IPTG. On the other hand, the extracellular proteolytic enzyme secreted by undomesticated *B. amyloliquefaciens* hydrolyzes the protein of interest to varying degrees [35]. To reduce the degradation of α-amylase by extracellular proteases secreted by undomesticated *B. amyloliquefaciens* LB1ba02, we deleted the genes of *aprE*, *nprE*, and *wprA*. The extracellular secretion capacity of the knockout strain for α-amylase was significantly better than that of the wild strain.

## Conclusion

In this work, we adapted a fast and efficient CRISPR/Cas9n-AID base editing system and developed a single plasmid CRISPR/Cas9n genome editing system in *B. amyloliquefaciens* LB1ba02. The CRISPR/Cas9n-AID single-base editing system can achieve multiple site editing simultaneously, and the CRISPR/Cas9n system can achieve rapid gene knockout and integration. In addition, we modified the wild strain LB1ba02 through these technologies and obtained a mutant strain LB1ba02△4 with stronger amylase secretion. Compared with LB1ba02, the mutant LB1ba02△4 secreted 1.25-fold more AmyX into the medium. The single plasmid CRISPR/Cas9n-AID and CRISPR/Cas9n systems developed in this work expand the range of the genetic manipulation of *B. amyloliquefaciens.*

## Materials and methods

### Strains and plasmids

All bacterial strains and plasmids used in this study are described in Table 1. *E.coli* Mach1-T1 was used for plasmid construction. The *dam-* and *dcm-* deficient *E. coli* strain JM110 was used for plasmid demethylation. The undomesticated *B. amyloliquefaciens* LB1ba02 was used for genetic manipulation and secretion of mesophilic α-amylase. Plasmids pwtCas9-bacteria and pgRNA-bacteria were purchased from Huayueyang Biotech (Beijing, China) Co., Ltd.

**Table 1:** Strains and plasmids used in this study

Strains and plasmids	Characteristics	Reference
Strains		
E.coli Mach1-T1	F- φ80(lacZ)ΔM15 ΔlacX74 hsdR(rK- mK +) ΔrecA1398 endA1 tonA	Huayueyang Bio
E.coli JM110	The dam- and dcm- deficient strain	Huayueyang Bio
B.amyloliquefaciens LB1ba02	Wild type	Preserved in our lab
B.amyloliquefaciens LB1ba02△pyrF	B.amyloliquefaciens LB1ba02 derivative, knock out the gene of pyrF	This work
B.amyloliquefaciens LB1ba02△bamHIR(GFP)	B.amyloliquefaciens LB1ba02 derivative, knock in the fluorescent protein GFP	This work
B.amyloliquefaciens LB1ba02△2	B.amyloliquefaciens LB1ba02 derivative, knock out the genes of aprE, and nprE	This work
B.amyloliquefaciens LB1ba02△3	B.amyloliquefaciens LB1ba02 derivative, knock out the genes of aprE, nprE, and wprA	This work
B.amyloliquefaciens LB1ba02△4	B.amyloliquefaciens LB1ba02 derivative, knock out the genes of aprE, nprE, wprA, and bamHIR	This work
Plasmids		
pwtCas9-bacteria	Amp, tetracycline repressor TetR, cas9 gene	Huayueyang Bio
pgRNA-bacteria	Amp, sgRNA plasmid, containing a chimera RNA 3'UTR, SptracrRNA terminator	Huayueyang Bio
pWSCas9n	Temperature-sensitive shuttle Cas9n expression plasmid, Kan (E.coli and B.amyloliquefaciens)	This work
pWSCas9n-AID	Cas9n fusion AID expression plasmid, Kan (E.coli and B.amyloliquefaciens)	This work
pWSCas9n-sgRNA-wprA	The CRISPR/Cas9n knockout plasmid, Cas9n, pE194ts, sgRNA, and donorDNA of wprA	This work
pWSCas9n-sgRNA-bamHIR	The CRISPR/Cas9n knockout plasmid, Cas9n, pE194ts, sgRNA, and donorDNA of bamHIR	This work
pWSCas9n-sgRNA-bamHIR(GFP)	The CRISPR/Cas9n fluorescent protein GFP integration plasmid	This work
pWSCas9n-AID-sgRNA-pyrF	CRISPR/Cas9n-AID Single base editing plasmid (pyrF)	This work
pWSCas9n-AID-sgRNA-abnA	CRISPR/Cas9n-AID Single base editing plasmid (abnA)	This work
pWSCas9n-AID-sgRNA-aprE-nprE-mpr	CRISPR/Cas9n-AID Single base editing plasmid (aprE-nprE-mpr)	This work

### Reagents and enzymes

Primer STAR Max DNA Polymerase was purchased from TaKaRa Biotech (Dalian, China) Co., Ltd. Green Taq™ Mix was purchased from Vazyme Biotech (Nanjing, China) Co., Ltd. Restriction enzymes, NEBuilder^®^ HiFi DNA Assembly Cloning Kit, and *Bam*HI DNA methyltransferase was purchased NEB (Beijing, China) Co., Ltd. HiPure Plasmid Micro Kit, HiPure DNA Pure Micro Kits, and HiPure Gel Pure DNA Mini Kit were purchased from Magen Bio (Shanghai, China) Co., Ltd. DNA-Sanger sequencing and DNA primer synthesis were performed by Sangon Biotech (Shanghai, China) Co., Ltd. Other chemicals were purchased from Aladdin Biochemical Technology (Shanghai, China) Co., Ltd.

### Media and culture conditions

Conventional plasmids construction, transformants identification, and seeds culture were performed in LB medium (10 g/L tryptone, 5 g/L yeast extract, and 10 g/L NaCl) at 37 °C, 220 rpm, adding antibiotics of different concentrations (ampicillin 100 μg/mL, kanamycin 50 μg/mL) as required. M9 medium: 1 g/L NH_4_Cl, 3 g/L KH_2_PO_4_, and 6 g/L Na_2_HPO_4_), 2 mM MgSO_4_, 0.4% (w/v) glucose, 0.0005% (w/v) thiamine, and 2% (w/v) agar for solidification. When required, the M9 medium was supplemented with 2 mM uracil and 1 mg/mL 5-FOA [44]. A single colony of *B. amyloliquefaciens* from the plate was seeded in LB liquid media (50 mL medium/250 mL Erlenmeyer flask) and incubated at 37 °C for 12 h at 220 rpm. The preculture was then inoculated into fermentation medium (100 mL fermentation medium/500 mL baffled flasks) with 2% (v/v) inoculation amount, 37 ℃, 220 rpm. The fermentation medium contained 6.5% corn flour, 4% soybean cake powder, 0.4% disodium hydrogen phosphate, 0.03% potassium dihydrogen phosphate, 0.05% high- temperature amylase, pH 7.3–7.5. The supernatant of the fermentation broth was centrifuged to measure the α-amylase activity.

### Cell growth curve determination

The tested strains were cultured in LB liquid medium overnight, then inoculated into LB liquid media (50 mL medium/250 mL Erlenmeyer flask) with 2% (v/v) inoculation amount and incubated at 37 °C, 220 rpm. The optical density at 600 nm (OD600) of the bacteria was determined using a spectrophotometer (Eppendorf BioPhotometer) every 1 h. Three parallel tests were performed for each sample.

### Construction of the Cas9n protein inducible expression plasmid pWSCas9n

The Cas9n (D10A) protein DNA was derived from the plasmid pwtCas9-bacteria and amplified by the Cas9n-F/Cas9n-R primers. The P_*grac*_ promoter is an IPTG inducible promoter, derived from the *B. subtilis* expression plasmid pHT01 and amplified by the primers P_*grac*_-F/P_*grac*_-R. The sequence containing the *E. coli* replication origin (Ori), *Bacillus* temperature-sensitive replication origin (Rep pE194^ts^), and kanamycin resistance (Kan) were amplified by primers ori-F/rep-R. These three fragments were assembled by the NEBuilder^®^ HiFi DNA Assembly Cloning Kit (In-Fusion cloning) and named pWSCas9n, as shown in Additional file 1: Figure S6. To facilitate the subsequent insertion of sgRNA and donor DNA sequences, we introduced the restriction sites of *Xba*I, *Sal*I, and *Sma*I into pWSCas9n (The linkers at both ends of the plasmid pWSCas9n were added with homologous sequences containing the restriction sites of *Xba*I, *Sal*I, and *Sma*I when circularized by In-Fusion cloning). All primers used in this study are listed in Additional file 1: Table S1.

### Insertion of the sgRNA and donor DNA sequences for CRISPR/Cas9n

The homologous repair template upstream 400 bp and downstream 400 bp were amplified by primers up (*wprA*)-F/R and down (*wprA*)-F/R. The P_43_ (no RBS) promoter and sgRNA fragment were amplified by primers P_43_-F/P_43-_*wprA*-R and sgRNA-*wprA-*F/sgRNA-R. The 20 bp sgRNA sequence was added to the 5'end of primers P_43_-*wprA*-R and sgRNA-*wprA*-F, and inserted by overlapping PCR. These four DNA fragments were overlapped each other and inserted into the *Sma*I site of the backbone plasmid pWSCas9n by In-Fusion cloning to obtain an all-in-one knockout plasmid pWSCas9-sgRNA-*wprA*, as shown in Additional file 1: Figure S1. To obtain the knockout plasmid pWSCas9-sgRNA-*bamHIR*, the homologous repair template upstream 400 bp and downstream 400 bp were amplified by primers up (*bamHIR*)-F/R and down (*bamHIR*)-F/R. The P_43_ (no RBS) promoter and sgRNA fragment were amplified by primers P_43_-F/P_43-_*bamHIR*-R and sgRNA-*bamHIR-*F/sgRNA-R. Similarly, these four fragments overlapped each other and were inserted into the *Sma*I site of the backbone plasmid pWSCas9n by In-Fusion cloning. To construct the integration plasmid pWSCas9-sgRNA-*bamHIR*(GFP), the P_*veg*_-GFP expression cassette was amplified by primers P_*veg*_-F/R and GFP-F/R, overlapped together and inserted into the plasmid pWSCas9-sgRNA-*bamHIR* between upstream and downstream (Fig. 5a). The plasmid template pWSCas9-sgRNA-*bamHIR* was amplified by primers VT-Cas9n-*bamHIR*-F/R.

Electrotransformation of *B. amyloliquefaciens* LB1ba02 was performed according to the method of Zakataeva et al. [10]. The competent cells added with plasmid DNA were exposed to a single electrical pulse by an Eppendorf Eporator under the condition of 12.5 kV/cm field strength for 4.5–6 ms.

### Construction of base editing plasmids

The AID was fused with pWSCas9n by primers AID-F/R and pWSCas9n-F/R to construct a base editing backbone plasmid pWSCas9n-AID (Additional file 1: Figure S7). The transcription of sgRNA is under the P_43_ (no RBS) promoter, which were amplified by primers P_43_-F/R and sgRNA-F/R and inserted into the *Sma*I restriction site, named pWSCas9n-AID-sgRNA (Fig. 1a). When replacing the 20 bp sgRNA sequence, an inverse PCR is performed to amplify the entire plasmid and then circularized by In-Fusion cloning. To construct the base editing plasmid pWSCas9n-AID-sgRNA-*pyrF*, the plasmid template pWSCas9n-AID-sgRNA was amplified by reverse primers sgRNA-*pyrF*-F and P_43_-*pyrF*-R; subsequently, the amplified fragment was circularized by In-Fusion cloning and transformed into *E. coli* Mach1-T1. The plasmid pWSCas9n-AID-sgRNA-*pyrF* was obtained after DNA sequencing. Similarly, the base editing plasmid pWSCas9n-AID-sgRNA-*abnA* was constructed by reverse primers sgRNA-*abnA*-F and P_43_-*abnA*-R.

To construct the three loci base editing plasmid pWSCas9n-AID-sgRNA-*aprE-nprE-mpr*, the P_43_-sgRNA-*mpr* expression cassette was amplified by primers P_43_-*mpr*-F/R and sgRNA-*mpr*-F/R; the P_43_-sgRNA-*nprE* expression cassette was amplified by primers P_43_-*nprE*-F/R and sgRNA-*nprE*-F/R; the P_43_-sgRNA-*aprE* expression cassette was amplified by primers P_43_-*aprE*-F/R and sgRNA-*aprE*-F/R. These six DNA fragments were overlapped each other and inserted into the *Xba*I site of the backbone plasmid pWSCas9n-AID by In-Fusion cloning to obtain the edited plasmid pWSCas9n-AID-sgRNA-*aprE-nprE-mpr* (Fig. 3a).

### The experimental procedure of CRISPR/Cas9n-AID base editing

The base-editing plasmid pWSCas9n-AID-sgRNA was transformed into *E. coli* JM110 for demethylation and treated with *Bam*HI methylase. Then the *Bam*HI-methylated pWSCas9n-AID-sgRNA was transformed into *B. amyloliquefaciens* LB1ba02, spread on kanamycin containing 50 μg/mL LB plate and cultured at 30 °C for 12–16 h. After the transformants on LB plate were verified by colony PCR, the correct single transformant containing the plasmid pWSCas9n-AID-sgRNA was induced in a liquid medium with 50 μg/mL kanamycin and 100 μM IPTG at 30 °C, 220 rpm for 24 h. Then the cell suspensions were diluted to 10^–5^ and spread on the LB plate at 37 °C for 12 h. Finally, 15 single colonies were randomly selected for PCR sequencing verification to represent the overall editing efficiency. The mutant edited successfully was cultured in a liquid medium at 42 °C for 12 h to lose the intracellular plasmid and achieve a trace-free gene knockout.

### Fluorescence assays

The host with the integrated green fluorescent protein, *B. amyloliquefaciens*△*bamHIR* (GFP), was inoculated into a 24-well plate and cultured for 24 h 37 °C and 220 rpm. 1 mL of cultured cells were centrifuged at 10,000 rpm for 2 min, washed 3 times with 1 × TE buffer (pH 8.0). Cells were resuspended in 1 mL of TE buffer and then 200 μL were transferred to a 96-well microtiter plate, and a Tecan Infinite M200 microplate reader detected the fluorescence. Fluorescence program: excitation 490 nm, emission 530 nm, gain value 100. Cell density detection was performed at 600 nm. The fluorescence of the control strain without GFP (CF) was used for correction, and the relative fluorescence intensities (FI) were calculated by [45].

$$ {\text{FI}}_{{{\text{corrected}}}} = {\text{ FI}}/{\text{OD}}_{{{600} \, ({\text{FI-TE}})}}- {\text{CF}}/{\text{OD}}_{{{600} \, ({\text{CF-TE}})}} $$

### Detection of α-amylase activity

1% soluble starch solution was used as the substrate, and the solvent was phosphate buffer (50 mM, pH 6.0). In the reaction system, 350 μL of the substrate were added to 50 μL of diluted enzyme solution, and the reaction was carried out at 60 ℃ for 10 min. To terminate the reaction, 800 μL of DNS (3,5-Dinitrosalicylic acid) solution were added, and the reaction was heated in a boiling water bath for 5 min. After a short centrifugation, 200 μL were taken to determine the absorbance value (540 nm). The enzyme solution inactivated by boiling for 5 min was used as a control. The means of three independent technical replicates were selected. One unit of activity was defined as the amount of enzyme required to release 1 μmol maltose per minute (U/mL). The equation of the maltose standard curve and enzyme activity conversion were shown in the additional material Additional file 1: Figure S5.

### Statistical analysis

All results were presented as the mean ± standard deviation. Statistical analyses were determined by 2-tailed Student's t-test between two groups. Statistical significance was expressed as * for *P* < 0.05 and ** for *P* < 0.01.

## Supplementary Information

**Additional file 1:** **Figure S1.** The construction process of an all-in-one temperature-sensitive knockout plasmid pWSCas9n-sgRNA-*bamHIR*. Backbone plasmid pWSCas9n containing the *E. coli* replication origin, the temperature-sensitive replication origin rep pE194^ts^, and a kanamycin resistance gene, Cas9n under the control of IPTG-inducible promoter P_*grac*_. The sgRNA transcribed from the *Bacillus subtilis* promoter P_43_, and donor DNA was used for homology repair of SSB. **Figure S2. **Editing efficiency test of the extracellaluar protease gene* wprA* by the single plasmid CRISPR/Cas9n system. Before knockout: 1209 bp. After knockout: 824 bp. **Figure S3. **The plasmid pBEP43 (demethylation first) with a *Bam*HI restriction site was transformed into LB1ba02 and LB1ba02△4. (a) Map of the plasmid pBEP43. (b)The transformation plate for LB1ba02△4/ pBEP43. (c) The transformation plate for LB1ba02/ pBEP43. **Figure S4. **The curing efficiency of the single temperature sensitive plasmid pWSCas9n-sgRNA-*bamHIR*. **Figure S5. **The maltose standard curve. **Figure S6.** Map of the plasmid pWSCas9n. **Figure S7.** Map of the plasmid pWSCas9n-AID. **Figure S8. **Editing efficiency determination of cytosine at the −15 to −20 positions. **Figure S9. **Editing efficiency determination of five consecutive cytosines (5Cs) at the −16 to −20 positions. **Table S1. **Primers used in this study

## Data Availability

All data generated or analysed during this study are included in this published article [and its additional information files].

## Abbreviations

CRISPR: Clustered regularly interspaced short palindromic repeats
GRAS: Generally recognized as food safe
5-FOA: 5-Fluoroorotic acid
U: uridine
5-FU: 5-Fluorouracil
DSBs: Double-strand breaks
AID: Activation-induced cytidine deaminase
RBS: Ribosome binding site
DNS: 3,5-Dinitrosalicylic acid
5Cs: Five consecutive cytosines
