# Supplementary material for: Development and application of a fast and efficient CRISPR-based genetic toolkit in Bacillus amyloliquefaciens LB1ba02
Source: Microb Cell Fact. 2022 May 28;21:99. doi: 10.1186/s12934-022-01832-2 (PMC9148480; doi:10.1186/s12934-022-01832-2)
Supplement: Supplementary file 1 — Additional file 1: Figure S1. The construction process of an all-in-one temperature-sensitive knockout plasmid pWSCas9n-sgRNA-bamHIR. Backbone plasmid pWSCas9n containing the E. coli replication origin, the temperature-sensitive replication origin rep pE194ts, and a kanamycin resistance gene, Cas9n under the control of IPTG-inducible promoter Pgrac. The sgRNA transcribed from the Bacillus subtilis promoter P43, and donor DNA was used for homology repair of SSB. Figure S2. Editing efficiency test of the extracellaluar protease gene wprA by the single plasmid CRISPR/Cas9n system. Before knockout: 1209 bp. After knockout: 824 bp. Figure S3. The plasmid pBEP43 (demethylation first) with a BamHI restriction site was transformed into LB1ba02 and LB1ba02△4. (a) Map of the plasmid pBEP43. (b)The transformation plate for LB1ba02△4/ pBEP43. (c) The transformation plate for LB1ba02/ pBEP43. Figure S4. The curing efficiency of the single temperature sensitive plasmid pWSCas9n-sgRNA-bamHIR. Figure S5. The maltose standard curve. Figure S6. Map of the plasmid pWSCas9n. Figure S7. Map of the plasmid pWSCas9n-AID. Figure S8. Editing efficiency determination of cytosine at the −15 to −20 positions. Figure S9. Editing efficiency determination of five consecutive cytosines (5Cs) at the −16 to −20 positions. Table S1. Primers used in this study [file 12934_2022_1832_MOESM1_ESM.docx]

Development and Application of a Fast and Efficient CRISPR-based Genetic Toolkit in Bacillus amyloliquefaciens LB1ba02

**Qinglong Xin, Yudan Chen, Qianlin Chen, Bin Wang*, Li Pan ***

Additional Material File 1

#
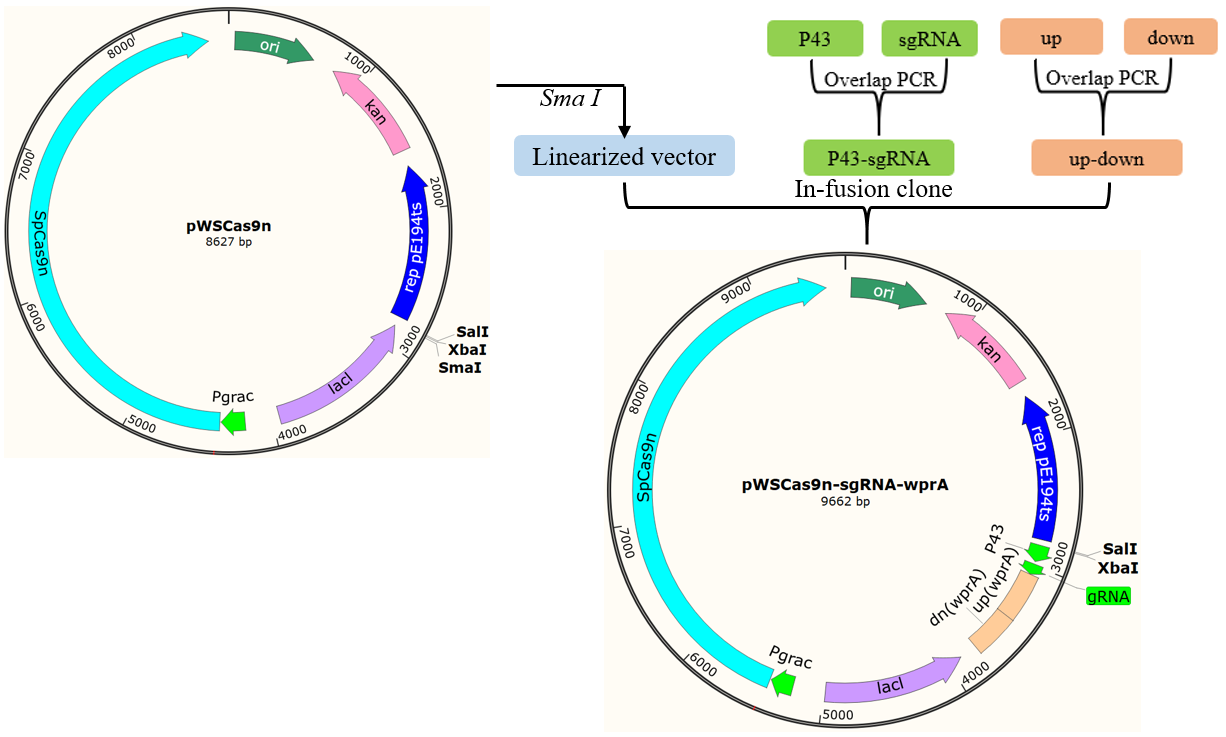


**Fig.S1.** The construction process of an all-in-one temperature-sensitive knockout plasmid pWSCas9n-sgRNA-*bamHIR*. Backbone plasmid pWSCas9n containing the *E. coli* replication origin, the temperature-sensitive replication origin rep pE194^ts^, and a kanamycin resistance gene, Cas9n under the control of IPTG-inducible promoter P*_grac_*. The sgRNA transcribed from the *Bacillus subtilis* promoter P_43_, and donor DNA was used for homology repair of SSB.


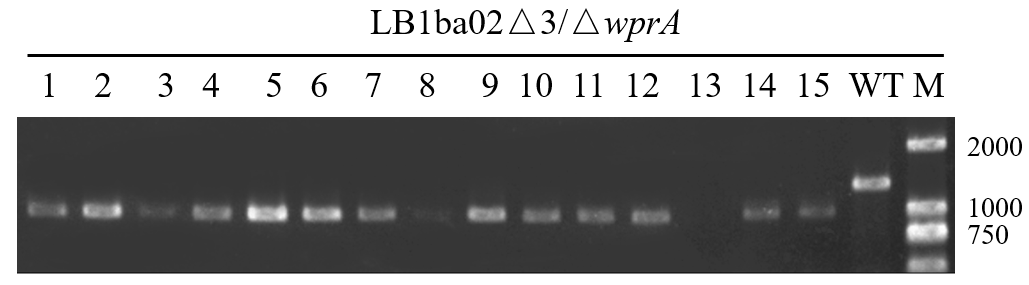


**Fig.S2.** Editing efficiency test of the [extracellaluar](javascript:;) [protease](javascript:;) gene *wprA* by the single plasmid CRISPR/Cas9n system. Before knockout: 1209 bp. After knockout: 824 bp.


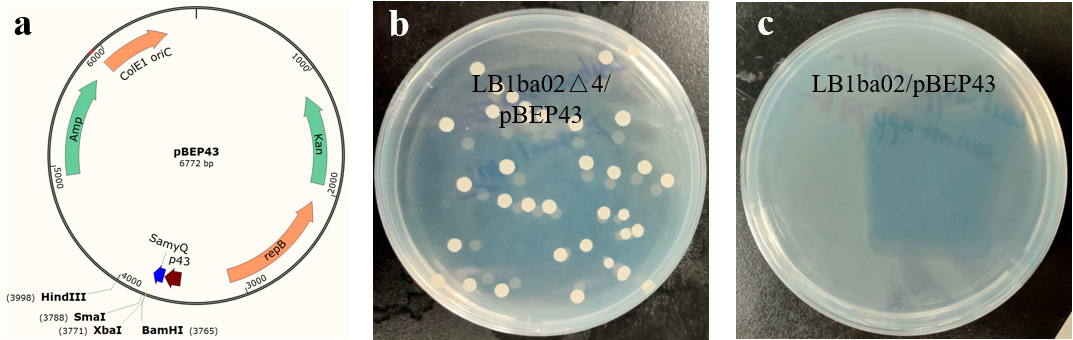


**Fig.S3.** The plasmid pBEP43 (demethylation first) with a *Bam*HI restriction site was transformed into LB1ba02 and LB1ba02△4. (a) Map of the plasmid pBEP43. (b)The transformation plate for LB1ba02△4/ pBEP43. (c) The transformation plate for LB1ba02/ pBEP43.


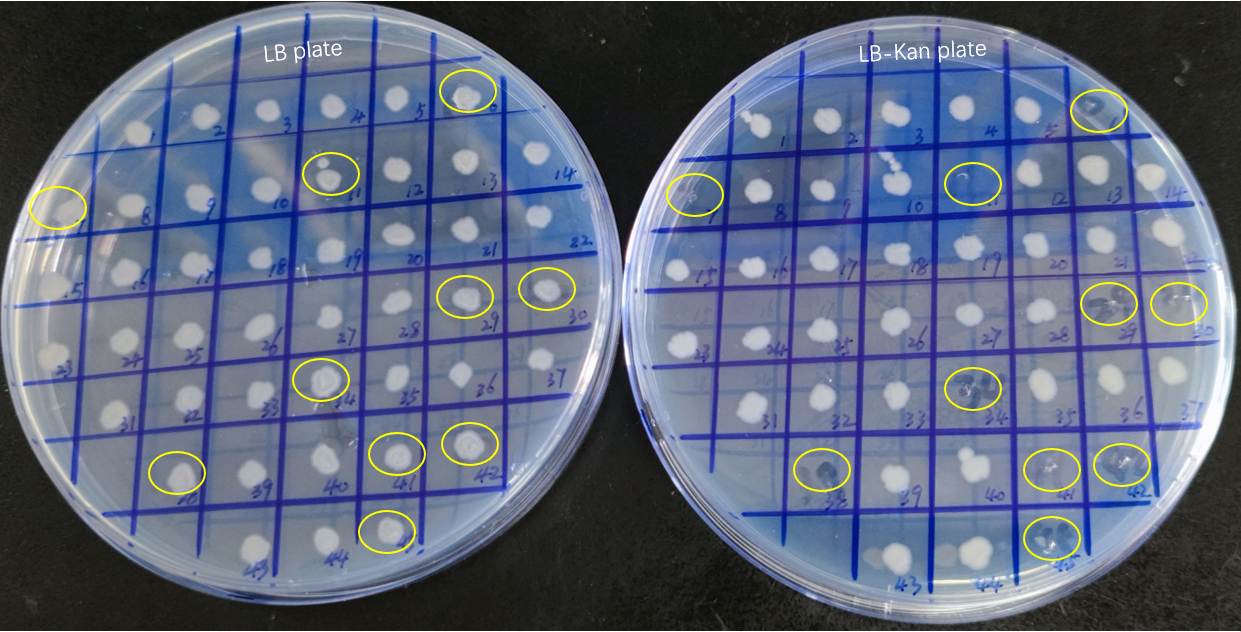


**Fig.S4.** The curing efficiency of the single temperature sensitive plasmid pWSCas9n-sgRNA-*bamHIR*


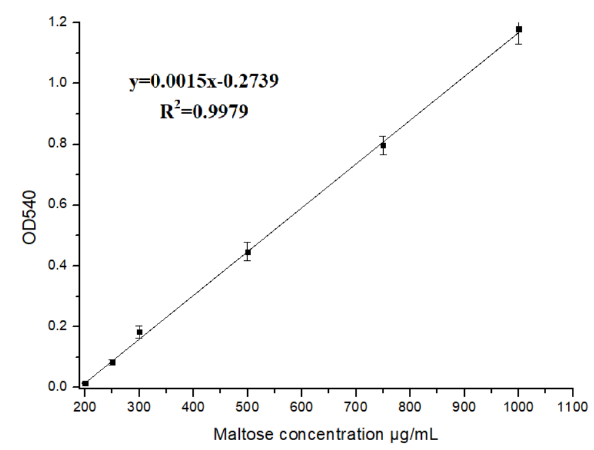


**Fig.S5.** The maltose standard curve

X=[(A+0.2739)/0.0015·N·24]/t·M

X: enzyme activity value (U/mL); A: absorbance value (540 nm); N: dilution times; t: reaction time (minutes); M: the molecular weight of maltose (360).

**Fig.S6.** Map of the plasmid pWSCas9n


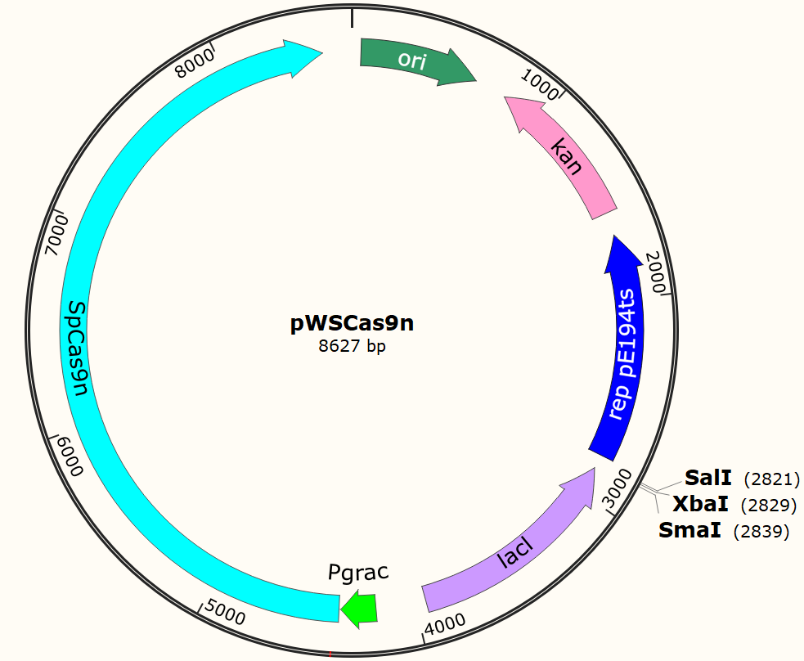


**Sequence of plasmid pWSCas9n**

cgttccactgagcgtcagaccccgtagaaaagatcaaaggatcttcttgagatcctttttttctgcgcgtaatctgctgcttgcaaacaaaaaaaccaccgctaccagcggtggtttgtttgccggatcaagagctaccaactctttttccgaaggtaactggcttcagcagagcgcagataccaaatactgtccttctagtgtagccgtagttaggccaccacttcaagaactctgtagcaccgcctacatacctcgctctgctaatcctgttaccagtggctgctgccagtggcgataagtcgtgtcttaccgggttggactcaagacgatagttaccggataaggcgcagcggtcgggctgaacggggggttcgtgcacacagcccagcttggagcgaacgacctacaccgaactgagatacctacagcgtgagctatgagaaagcgccacgcttcccgaagggagaaaggcggacaggtatccggtaagcggcagggtcggaacaggagagcgcacgagggagcttccagggggaaacgcctggtatctttatagtcctgtcgggtttcgccacctctgacttgagcgtcgatttttgtgatgctcgtcaggggggcggagcctatggaaaaacgccagcaacgcggcctttttacggttcctggccttttgctggccttttgctcacatgttctttcctgcgttatcccctgattctgtggataaccgtattaccgcctttgagtgagctgaaattatgaggggatctctcagagctcgaggtcatcgttcaaaatggtatgcgttttgacacatccactatatatccgtgtcgttctgtccactcctgaatcccattccagaaattctctagcgattccagaagtttctcagagtcggaaagttgaccagacattacgaactggcacagatggtcataacctgaaggaagatctgattgcttaactgcttcagttaagaccgaagcgctcgtcgtataacagatgcgatgatgcagaccaatcaacatggcacctgccattgctacctgcacagtcaaggatggtagaaatgttgtcggtccttgcacacgaatattacgccatttgcctgcatattcaaacagctcttctacgataagggcacaaatcgcatcgtggaacgtttgggcttctaccgatttagcagtttgatacactttctctaagtatccacctgaatcataaatcggcaaaatagagaaaaattgaccatgtgtaagcggccaatctgattccacctgagatgcataatctagtagaatctcttcgctatcaaaattcacttccaccttccactcaccggttgtccattcatggctgaactctgcttcctctgttgacatgacacacatcatctcaatatccgaatagggcccatcagtctgacgaccaagagagccataaacaccaatagccttaacatcatccccatatttatccaatattcgttccttaatttcatgaacaatcttcattctttcttctctagtcattattattggtccattcactattctcattcccttttcagataattttagatttgcttttctaaataagaatatttggagagcaccgttcttattcagctattaaacccattatatcgggtttttgaggggatttcaactgcagacacctaaattcaaaatctatcggtcagatttataccgatttgattttatatattcttgaataacatacgccgagttatcacataaaagcgggaaccaatcatcaaatttaaacttcattgcataatccattaaactcttaaattctacgattccttgttcatcaataaactcaatcatttctttaattaatttatatctatctgttgttgttttctttaataattcatcaacatctacaccgccataaactatcatatcttctttttgatatttaaatttattaggatcgtccatgtgaagcatatatctcacaagacctttcacacttcctgcaatctgcggaatagtcgcattcaattcttctgttaattatttttatctgttcataagatttattaccctcatacatcactagaatatgataatgctcttttttcatcctatcttctgtatcagtatccctatcatgtaatggagacactacaaattgaatgtgtaactcttttaaatactctaaccactcggcttttgctgattctggatataaaacaaatgtccaattacgtcctcttgaatttttcttgttttcagtttcttttattacattttcgctcatgatataataacggtgctaatacatttaacaaaatttagtcatagataggcagcatgccagtgctgtctatctttttttgtttaaaatgcaccgtattcctcctttgcatatttttttattagaataccggttgcatctgatttgctaatattatatttttctttgattctatttaatatctcattttcttctgttgtaagtcttaaagtaacagcaacttttttctcttcttttctatctacaaccatcactgtacctcccaacatctgtttttttcactttaacataaaaaacaaccttttaacattaaaaacccaatatttatttatttgtttggacaatggacaatggacacctaggggggaggtcgtagtacccccctatgttttctcccctaaataaccccaaaaatctaagaaaaaaagacctcaaaaaggtctttaattaacatctcaaatttcgcatttattccaatttcctttttgcgtgtgatgcgaattcttgaccgtgattagagaattggtcgactatctagatacccggggggccaataaggcctttctagtcactgcccgctttccagtcgggaaacctgtcgtgccagctgcattaatgaatcggccaacgcgcggggagaggcggtttgcgtattgggcgccagggtggtttttcttttcaccagtgagacgggcaacagctgattgcccttcaccgcctggccctgagagagttgcagcaagcggtccacgctggtttgccccagcaggcgaaaatcctgtttgatggtggttaacggcgggatataacatgagctgtcttcggtatcgtcgtatcccactaccgagatatccgcaccaacgcgcagcccggactcggtaatggcgcgcattgcgcccagcgccatctgatcgttggcaaccagcatcgcagtgggaacgatgccctcattcagcatttgcatggtttgttgaaaaccggacatggcactccagtcgccttcccgttccgctatcggctgaatttgattgcgagtgagatatttatgccagccagccagacgcagacgcgccgagacagaacttaatgggcccgctaacagcgcgatttgctggtgacccaatgcgaccagatgctccacgcccagtcgcgtaccgtcttcatgggagaaaataatactgttgatgggtgtctggtcagagacatcaagaaataacgccggaacattagtgcaggcagcttccacagcaatggcatcctggtcatccagcggatagttaatgatcagcccactgacgcgttgcgcgagaagattgtgcaccgccgttttacaggcttcgacgccgcttcgttctaccatcgacaccaccacgctggcacccagttgatcggcgcgagatttaatcgccgcgacaatttgcgacggcgcgtgcagggccagactggaggtggcaacgccaatcagcaacgactgtttgcccgccagttgttgtgccacgcggttgggaatgtaattcagctccgccatcgccgcttccactttttcccgcgttttcgcagaaacgtggctggcctggttcaccacgcgggaaacggtctgataagagacaccggcatactctgcgacatcgtataacgttactggtttcatcaaaatcgtctccctccgtttgaatatttgattgatcgtaaccagatgaagcactctttccactatccctacagtgttatggcttgaacaatcacgaaacaataattggtacgtacgatctttcagccgactcaaacatcaaatcttacaaatgtagtctttgaaagtattacatatgtaagatttaaatgcaaccgttttttcggaaggaaatgatgacctcgtttccaccggaattagcttggtaccagctattgtaacataatcggtacgggggtgaaaaagctaacggaaaagggagcggaaaagaatgatgtaagcgtgaaaaattttttatcttatcacttgaaattggaagggagattctttattataagaattgtggaattgtgagcggataacaattcccaattaaaggaggaaggatcctatggataagaaatactcaataggcttagctatcggcacaaatagcgtcggatgggcggtgatcactgatgaatataaggttccgtctaaaaagttcaaggttctgggaaatacagaccgccacagtatcaaaaaaaatcttataggggctcttttatttgacagtggagagacagcggaagcgactcgtctcaaacggacagctcgtagaaggtatacacgtcggaagaatcgtatttgttatctacaggagattttttcaaatgagatggcgaaagtagatgatagtttctttcatcgacttgaagagtcttttttggtggaagaagacaagaagcatgaacgtcatcctatttttggaaatatagtagatgaagttgcttatcatgagaaatatccaactatctatcatctgcgaaaaaaattggtagattctactgataaagcggatttgcgcttaatctatttggccttagcgcatatgattaagtttcgtggtcattttttgattgagggagatttaaatcctgataatagtgatgtggacaaactatttatccagttggtacaaacctacaatcaattatttgaagaaaaccctattaacgcaagtggagtagatgctaaagcgattctttctgcacgattgagtaaatcaagacgattagaaaatctcattgctcagctccccggtgagaagaaaaatggcttatttgggaatctcattgctttgtcattgggtttgacccctaattttaaatcaaattttgatttggcagaagatgctaaattacagctttcaaaagatacttacgatgatgatttagataatttattggcgcaaattggagatcaatatgctgatttgtttttggcagctaagaatttatcagatgctattttactttcagatatcctaagagtaaatactgaaataactaaggctcccctatcagcttcaatgattaaacgctacgatgaacatcatcaagacttgactcttttaaaagctttagttcgacaacaacttccagaaaagtataaagaaatcttttttgatcaatcaaaaaacggatatgcaggttatattgatgggggagctagccaagaagaattttataaatttatcaaaccaattttagaaaaaatggatggtactgaggaattattggtgaaactaaatcgtgaagatttgctgcgcaagcaacggacctttgacaacggctctattccccatcaaattcacttgggtgagctgcatgctattttgagaagacaagaagacttttatccatttttaaaagacaatcgtgagaagattgaaaaaatcttgacttttcgaattccttattatgttggtccattggcgcgtggcaatagtcgttttgcatggatgactcggaagtctgaagaaacaattaccccatggaattttgaagaagttgtcgataaaggtgcttcagctcaatcatttattgaacgcatgacaaactttgataaaaatcttccaaatgaaaaagtactaccaaaacatagtttgctttatgagtattttacggtttataacgaattgacaaaggtcaaatatgttactgaaggaatgcgaaaaccagcatttctttcaggtgaacagaagaaagccattgttgatttactcttcaaaacaaatcgaaaagtaaccgttaagcaattaaaagaagattatttcaaaaaaatagaatgttttgatagtgttgaaatttcaggagttgaagatagatttaatgcttcattaggtacctaccatgatttgctaaaaattattaaagataaagattttttggataatgaagaaaatgaagatatcttagaggatattgttttaacattgaccttatttgaagatagggagatgattgaggaaagacttaaaacatatgctcacctctttgatgataaggtgatgaaacagcttaaacgtcgccgttatactggttggggacgtttgtctcgaaaattgattaatggtattagggataagcaatctggcaaaacaatattagattttttgaaatcagatggttttgccaatcgcaattttatgcagctgatccatgatgatagtttgacatttaaagaagacattcaaaaagcacaagtgtctggacaaggcgatagtttacatgaacatattgcaaatttagctggtagccctgctattaaaaaaggtattttacagactgtaaaagttgttgatgaattggtcaaagtaatggggcggcataagccagaaaatatcgttattgaaatggcacgtgaaaatcagacaactcaaaagggccagaaaaattcgcgagagcgtatgaaacgaatcgaagaaggtatcaaagaattaggaagtcagattcttaaagagcatcctgttgaaaatactcaattgcaaaatgaaaagctctatctctattatctccaaaatggaagagacatgtatgtggaccaagaattagatattaatcgtttaagtgattatgatgtcgatcacattgttccacaaagtttccttaaagacgattcaatagacaataaggtcttaacgcgttctgataaaaatcgtggtaaatcggataacgttccaagtgaagaagtagtcaaaaagatgaaaaactattggagacaacttctaaacgccaagttaatcactcaacgtaagtttgataatttaacgaaagctgaacgtggaggtttgagtgaacttgataaagctggttttatcaaacgccaattggttgaaactcgccaaatcactaagcatgtggcacaaattttggatagtcgcatgaatactaaatacgatgaaaatgataaacttattcgagaggttaaagtgattaccttaaaatctaaattagtttctgacttccgaaaagatttccaattctataaagtacgtgagattaacaattaccatcatgcccatgatgcgtatctaaatgccgtcgttggaactgctttgattaagaaatatccaaaacttgaatcggagtttgtctatggtgattataaagtttatgatgttcgtaaaatgattgctaagtctgagcaagaaataggcaaagcaaccgcaaaatatttcttttactctaatatcatgaacttcttcaaaacagaaattacacttgcaaatggagagattcgcaaacgccctctaatcgaaactaatggggaaactggagaaattgtctgggataaagggcgagattttgccacagtgcgcaaagtattgtccatgccccaagtcaatattgtcaagaaaacagaagtacagacaggcggattctccaaggagtcaattttaccaaaaagaaattcggacaagcttattgctcgtaaaaaagactgggatccaaaaaaatatggtggttttgatagtccaacggtagcttattcagtcctagtggttgctaaggtggaaaaagggaaatcgaagaagttaaaatccgttaaagagttactagggatcacaattatggaaagaagttcctttgaaaaaaatccgattgactttttagaagctaaaggatataaggaagttaaaaaagacttaatcattaaactacctaaatatagtctttttgagttagaaaacggtcgtaaacggatgctggctagtgccggagaattacaaaaaggaaatgagctggctctgccaagcaaatatgtgaattttttatatttagctagtcattatgaaaagttgaagggtagtccagaagataacgaacaaaaacaattgtttgtggagcagcataagcattatttagatgagattattgagcaaatcagtgaattttctaagcgtgttattttagcagatgccaatttagataaagttcttagtgcatataacaaacatagagacaaaccaatacgtgaacaagcagaaaatattattcatttatttacgttgacgaatcttggagctcccgctgcttttaaatattttgatacaacaattgatcgtaaacgatatacgtctacaaaagaagttttagatgccactcttatccatcaatccatcactggtctttatgaaacacgcattgatttgagtcagctaggaggtgactaactcgagtaaggatctccaggcatcaaataaaacgaaaggctcagtcgaaagactgggcctttcgttttatctgttgtttgtcggtgaacgctctctactagagtcacactggctcaccttcgggtgggcctttctgcgtttata

**Fig.S7.** Map of the plasmid pWSCas9n-AID


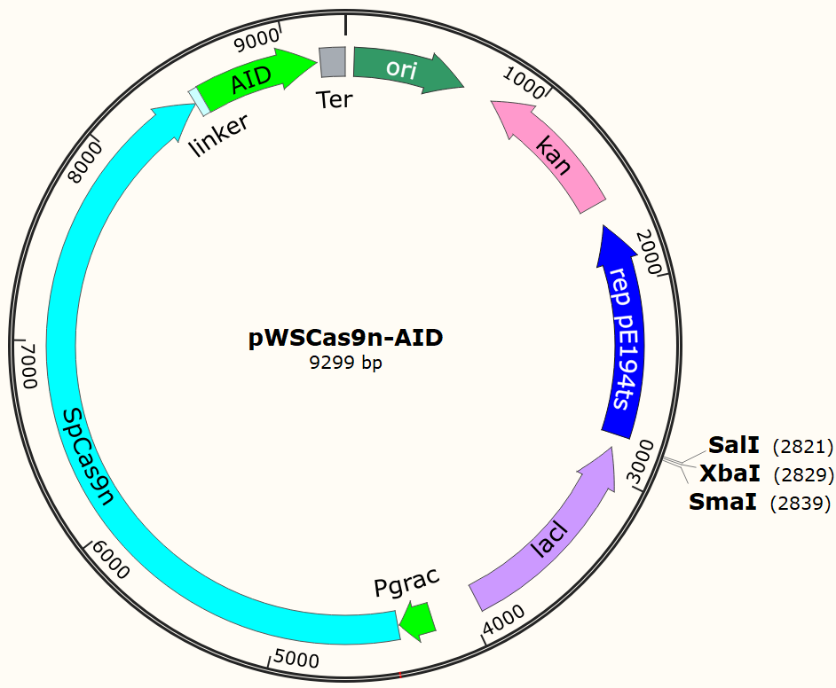


**Sequence of Linker-AID**

Agcggcggaggcggatcaggcggaggcggaagcggcggaggcggatcaacggatgcggaatatgttagaattcatgaaaaactggatatctatacattcaaaaaacagtttttcaacaacaaaaaatcagtttcacatcgctgctatgtcctgtttgaacttaaaagacgcggcgaacggagagcgtgcttttggggatatgccgtcaataaaccgcagagcggcacggaaagaggaattcatgccgaaatttttagcatcagaaaagtggaagaatatcttcgcgataatccgggccagtttacaattaactggtattcaagctggagcccgtgcgccgattgcgcagaaaaaatcctggaatggtataatcaggaacttcggggcaacggacatacgctgaaaatttgggcgtgcaaactttattatgagaaaaatgcccgcaaccagatcggcctgtggaatcttcgggataacggcgtgggactgaatgtcatggtcagcgaacattaccagtgctgcagaaaaatcttcatccagagctcacataaccagcttaatgaaaaccggtggctggaaaaaacacttaaaagagcggaaaaaagaagaagcgaactgtcaattatgatccaggtgaaaatccttcatacgacaaaatcaccggccgtttaa


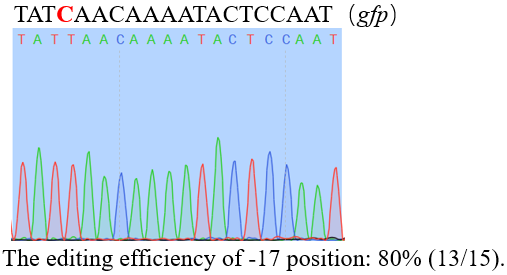

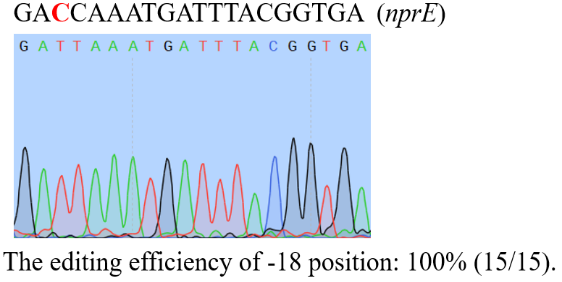

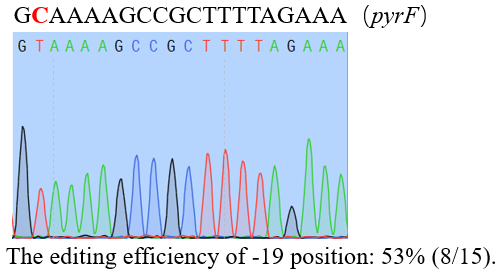

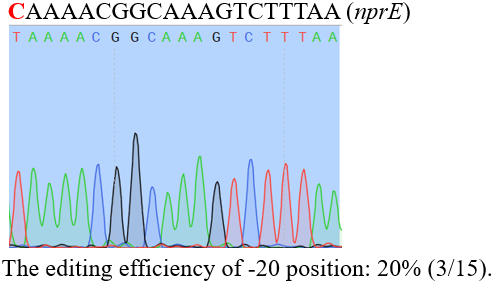
**Fig.S8.** Editing efficiency determination of cytosine at the −15 to −20 positions.


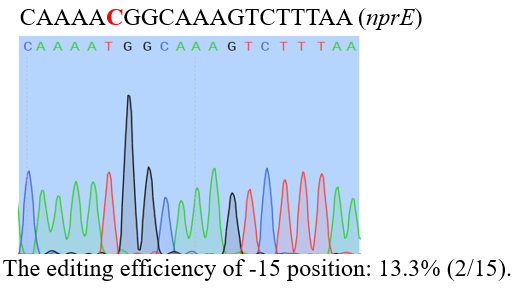

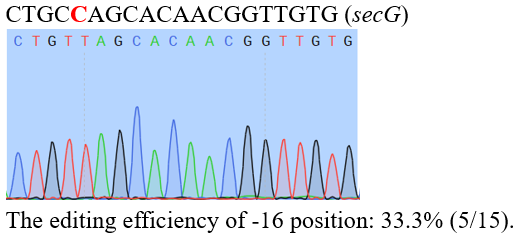


*The means of three independent technical replicates are presented; the final value is the average of three independent technical replicates.

**Fig.S9.** Editing efficiency determination of five consecutive cytosines (5Cs) at the −16 to −20 positions.


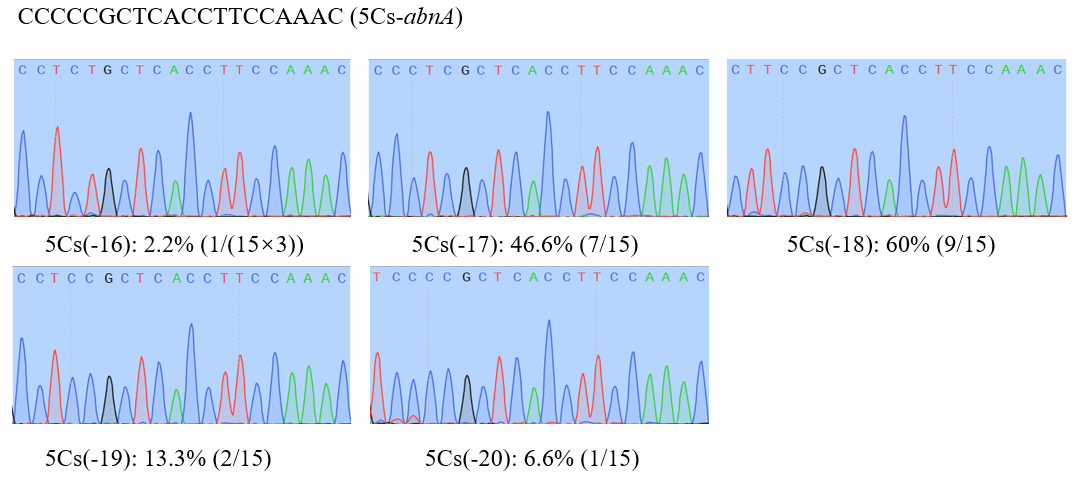


*The means of three independent technical replicates are presented; the final value is the average of three independent technical replicates.

**Table. S1. Primers used in this study**

| **Primers** | **Sequence(5'-3')** |  |
| --- | --- | --- |
| Cas9n-F | attaaaggaggaaggatcctatggataagaaatactcaataggctt |  |
| Cas9n-R | tataaacgcagaaaggcccaccc |  |
| P*_grac_*-F | gtcgactatctagatacccggggggccaataaggcctttctagtc |  |
| P*_grac_*-R | aggatccttcctcctttaattggg |  |
| ori-F | tgggcctttctgcgtttatacgttccactgagcgtcagacc |  |
| rep-R | cgggtatctagatagtcgaccaat |  |
| P_43_-F | ggtcgactatctagatacccggtttacttatttttttgccaaagct |  |
| P_43_-R | ctataatggtaccgctatcactttat |  |
| P_43-_*wprA*-R | ttctggtctttcggcatgtcctataatggtaccgctatcactttat |  |
| P_43-_*bamHIR*-R | acacgtttaagttcactgttctataatggtaccgctatcactttat |  |
| P_43_-*pyrF*-R | tttctaaaagcggcttttgcctataatggtaccgctatcacttt |  |
| P_43_-*abnA*-R | gtttggaaggtgagcgggggctataatggtaccgctatcacttt |  |
| sgRNA-F | tgatagcggtaccattatag gttttagagctagaaatagcaagtt |  |
| sgRNA-R | aaaggccttattggccccccaaaaaagcaccgactcggtgcc |  |
| sgRNA-*pyrF*-F | gcaaaagccgcttttagaaagttttagagctagaaatagcaagtt |  |
| sgRNA-*abnA*-F | cccccgctcaccttccaaacgttttagagctagaaatagcaagtt |  |
| sgRNA-*aprE* | ttccaagacaacaactctca |  |
| sgRNA-*mpr* | accaacagttccagtccggc |  |
| sgRNA-*nprE* | gaccaaatgatttacggtga |  |
| sgRNA-*bamHIR-*F | aacagtgaacttaaacgtgt gttttagagctagaaatagcaagtt |  |
| sgRNA-*wprA-*F | gacatgccgaaagaccagaa gttttagagctagaaatagcaagtt |  |
| up(*wprA*)-F | ggcaccgagtcggtgctttttt caaaccccgactggcaaagagt |  |
| up(*wprA*)-R | ggtctgaagcttgctttttgcag |  |
| dn(*wprA*)-F | aaaaagcaagcttcagaccgggaagcttactgacacgctcattgc |  |
| dn(*wprA*)-R | aaaggccttattggccccccctcccccaaggctcaggttaat |  |
| VF-(*wprA*)-F | ttgcggacgatccgtgatcaag |  |
| VF-(*wprA*)-R | ttcagagcgtattcaagcactcg |  |
| up(*bamHIR*)-F | ggcaccgagtcggtgcttttttctagcttttgaatgtgacctacac |  |
| up(*bamHIR*)-R | aattggtactacaccgttacaattc |  |
| dn(*bamHIR*)-F | gaattgtaacggtgtagtaccaatttgctgaggcttataattctaatgtc |  |
| dn(*bamHIR*)-R | aaaggccttattggccccccttaactatttcagttgcaacgttgtt |  |
| VF-(*pyrF*)-F | ggcgttgatctcgtcaatgtcc |  |
| VF-(*pyrF*)-R | acttgatcgtcagatgcgtcact |  |
| VF-(*wprA*)-F | ttgcggacgatccgtgatcaag |  |
| VF-(*wprA*)-R | ttcagagcgtattcaagcactcg |  |
| VF-(*bamHI*)-F | cgtcaattgttatcaggaggtac |  |
| VF-(*bamHI*)-R | ttgcccgagaggacgataaactc |  |
| VF-(*nprE*)-F | cgctgagaatcctcagcttaaag |  |
| VF-(*nprE*)-R | cacgatagtaaatctgctccgct |  |
| VF-(*mpr*)-F | agacatctgtttcaagcagcggt |  |
| VF-(*mpr*)-R | gatctgactccgccgctttcat |  |
| VF-(*aprE*)-F | ggatcagtttgctgtttgctttag |  |
| VF-(*aprE*)-R | cgcctgtacgttgatcagccc |  |
| VF-(*abnA*)-F | caaacgcttgatccgcttttcgt |  |
| VF-(*abnA*)-R | tgttgatgaagcgagaccgattg |  |
| pWSCas9n-F | caaaatcaccggccgtttaaccaggcatcaaataaaacgaaagg |  |
| pWSCas9n-R | gtcacctcctagctgactcaaat |  |
| AID-F | tgagtcagctaggaggtgacagcggcggaggcggatcag |  |
| AID-R | ttaaacggccggtgattttgtcg |  |
| Pveg-F | gtaacggtgtagtaccaattcagttgaaaacctgcataggagag |  |
| Pveg-R | tgcatccacctcactacatttattg |  |
| GFP-F | aatgtagtgaggtggatgcaatgagtaaaggagaagaacttttcac |  |
| GFP-R | tctagatagttatttgtatagttcatc |  |
| VT-Cas9n-*bamHIR*-F | tatacaaataactatctagatgctgaggcttataattctaatgtc |  |
| VT-Cas9n-*bamHIR*-R | aattggtactacaccgttacaattc |  |
| P43-*mpr*-F | gtgattagagaattggtcgactatggtttacttatttttttgccaaagc |  |
| P43-*mpr*-R | gccggactggaactgttggtctataatggtaccgctatcacttt |  |
| sgRNA-*mpr*-F | accaacagttccagtccggcgttttagagctagaaatagcaagtt |  |
| sgRNA-*mpr*-R | gatttttaaacgagcacgagagcaaaaaaagcaccgactcggtgcc |  |
| P43-*nprE*-F | tgctctcgtgctcgtttaaaaatcggtttacttatttttttgccaaagc |  |
| P43-*nprE*-R | tcaccgtaaatcatttggtcctataatggtaccgctatcacttt |  |
| sgRNA-*nprE*-F | gaccaaatgatttacggtgagttttagagctagaaatagcaagtt |  |
| sgRNA-*nprE*-R | ctggctttcggtaagctagacaaaaaaaagcaccgactcggtgcc |  |
| P43-*aprE*-F | ttgtctagcttaccgaaagccagatacccggtttacttatttttttgcc |  |
| P43-*aprE*-R | tgagagttgttgtcttggaactataatggtaccgctatcacttt |  |
| sgRNA-*aprE*-F | ttccaagacaacaactctcagttttagagctagaaatagcaagtt |  |
| sgRNA-*aprE*-R | cttattggccccccgggtataaaaaagcaccgactcggtgcc |  |
| * The base marked in red represent the 20 bp sgRNA sequence. | |  |
|  |  |  |
